# A Hardware-Friendly Optical Flow-Based Time-to-Collision Estimation Algorithm

**DOI:** 10.3390/s19040807

**Published:** 2019-02-16

**Authors:** Cong Shi, Zhuoran Dong, Shrinivas Pundlik, Gang Luo

**Affiliations:** 1 School of Microelectronics and Communication Engineering, Chongqing University, Chongqing 400044, China; 2 Schepens Eye Research Institute, Massachusetts Eye and Ear, Harvard Medical School, Boston, MA 02114, USA; shrinivas_pundlik@meei.harvard.edu (S.P.); gang_luo@meei.harvard.edu (G.L.); 3 Viterbi School of Engineering, University of Southern California, Los Angeles, CA 90089, USA; zhuoran@usc.edu

**Keywords:** time-to-collision, optical flow, motion estimation, motion energy, spatiotemporal energy, biological visual features

## Abstract

This work proposes a hardware-friendly, dense optical flow-based Time-to-Collision (TTC) estimation algorithm intended to be deployed on smart video sensors for collision avoidance. The algorithm optimized for hardware first extracts biological visual motion features (motion energies), and then utilizes a Random Forests regressor to predict robust and dense optical flow. Finally, TTC is reliably estimated from the divergence of the optical flow field. This algorithm involves only feed-forward data flows with simple pixel-level operations, and hence has inherent parallelism for hardware acceleration. The algorithm offers good scalability, allowing for flexible tradeoffs among estimation accuracy, processing speed and hardware resource. Experimental evaluation shows that the accuracy of the optical flow estimation is improved due to the use of Random Forests compared to existing voting-based approaches. Furthermore, results show that estimated TTC values by the algorithm closely follow the ground truth. The specifics of the hardware design to implement the algorithm on a real-time embedded system are laid out.

## 1. Introduction

The concept of time-to-collision (TTC) is important for collision alerting/avoidance and finds applications in robot navigation [1,2], mobility assistive devices for visually impaired people [3,4], and driving safety [5]. The main advantage of using TTC is its relative simplicity. Unlike in collision warning systems based on range sensors (radar, laser, structured-light depth sensor), or stereo cameras that measure the distance to the obstacles by binocular matching, TTC computation, requiring only a monocular camera, can be done without knowing the physical distance to the objects [1,2,3,4,5,6,7,8,9,10,11,12]. Therefore, low cost smart video sensors for collision avoidance can be made feasible by employing TTC-based approaches. In this paper, we describe an algorithm for TTC computation with the intention of deploying it as a smart video sensor for collision avoidance.

Some of the desirable qualities for the TTC algorithm for smart video sensors are low cost and good scalability for embedded implementation, real-time performance, and dense pixel-wise output. At a high level, TTC computation generally involves a way of first quantifying some measure of change in the images of the monocular video stream and then converting this quantified change to a usable TTC value (say, via scaling). 

Existing TTC estimation approaches can be classified into three broad categories: feature-based (inferring TTC from the change of obstacle scale with sparse feature tracking or matching) [1,3,4,5,6,7,8], gradient-based (direct estimation from image intensity or spatiotemporal gradients) [2,9,10,11], and dense optical flow-based [12,13,14,15]. From the perspective of deployment on smart video sensors for our application, feature-based algorithms are not very suitable for efficient hardware implementation. While simple and elegant, gradient-based approaches are not robust to brightness changes [16] that are routinely experienced in real-world situations when the camera approaches an object. 

Compared to the other two kinds of approaches, the dense optical flow-based methods provide a pixel-level TTC map rather than individual object- or region-level TTC values and thus tend to be more desirable given the application, because the pixel-level TTC values can be flexibly processed by higher-level analyses. Given the multitude of dense optical flow algorithms [17], we could theoretically choose a highly accurate algorithm for implementation. However, most of the dense optical flow algorithms rely on computationally intensive iterative and/or global optimizers, preventing them from being implemented in resource-limited real-time embedded systems. Despite this, a particular class of optical flow algorithms based on bio-inspired motion features [13,18,19,20,21,22,23,24] has potential for hardware-friendly implementation. In particular, the extraction of bio-inspired motion energy features by spatiotemporal filters has good parallelism for hardware acceleration. Besides, the motion energy features are robust to noises as they are a distributed representation of the motion speed [14]. However, the 3D spatiotemporal filtering (X, Y and time) still requires a considerable amount of multiplication operations. Moreover, computation of the optical flow from the motion energy features was done either in a Bayesian inference manner [13,19] (which required computing the probability distribution of the optical flow from the motion energy features), or in a renormalization-and-weighted-average manner [20] (which involved a number of complicated division operations). Both methods involve large computational overhead and are difficult to be scalable for conventional embedded systems. 

Recently, approaches that partially optimize the motion energy-based optical flow computation for compact hardware implementation have been proposed [14,25], which decompose one 3D spatiotemporal filter for motion energy extraction into cascaded 1D spatial and temporal filters. Such complexity reduction from *O*(*N*^3^) to *O*(5*N*) (where *N* is the filter dimension, which is much larger than 5) can save a large amount of hardware resources, which is highly desirable for smart video sensor applications. 

In this paper, we propose a novel hardware-friendly TTC estimation algorithm based on the field divergence of optical flow derived from motion energy features. The main contribution and novelty of our work is the use of Random Forests (an ensemble of decision trees) [26,27] in computing optical flow from motion energy features. To our knowledge, this is the first time that computationally inexpensive machine learning tools have been applied to motion energy-based optical flow computation. Compared to previous work using dedicated weighted voting scheme [14,19] to model the Bayesian inference, the Random Forests method exhibits better adaptation to a larger speed range, and significantly reduces computational resource budget by eliminating all multiplication operations when inferring optical flow from the extracted motion energy. We demonstrate the effectiveness of our TTC computation algorithm in various image sequences with looming motion and discuss the feasibility of implementing it on a low-cost real-time custom hardware. 

This paper is organized as follows: Section 2 describes the details of our algorithm. Section 3 gives experimental results of optical flow and TTC estimation of the algorithm, including demonstrations of the advantages of random forests over voting schemes in optical flow accuracy. Section 4 discusses the feasibility of implementing the algorithm on low-cost real-time customized hardware for smart video sensors, and Section 5 concludes this work.

## 2. Proposed TTC Estimation Algorithm

**Figure 1:**
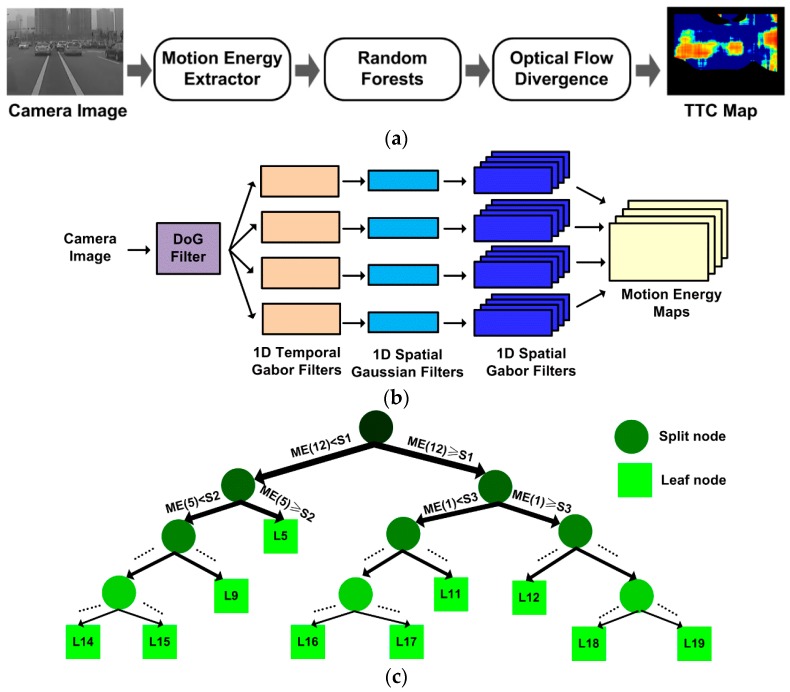
The proposed hardware-friendly TTC estimation algorithm based on optical flow. (**a**) Algorithm flow overview. (**b**) Spatiotemporal filters for motion energy extraction. (**c**) The structure of a trained regression tree in the random forests.

An overview of the proposed hardware-friendly TTC estimation algorithm is shown in Figure 1a. It contains three stages. First, the frame sequence of camera images is processed to extract the motion energy features, which are then synthesized into dense optical flow by Random Forests. Finally, the TTC map is estimated based on the field divergence of the optical flow. The details of the three stages are described in the following subsections. 

### 2.1. Biological Motion Energy Extraction

The procedure for motion energy feature extraction involves a Difference-of-Gaussian (DoG) filter and a bank of spatial and temporal Gabor filters (Figure 1b) that mimic the functions of retina ganglion cells and cortex V1 cells in primate visual systems, respectively [28]. The DoG filter removes near-zero spatial frequency band and the high spatial frequency noise that are detrimental to optical flow accuracy. Compared to the binary and ternary pre-processed images in [14,25], the DoG filtered image preserves a broader spatial frequency band leading to more accurate optical flow estimation. Next, the DoG filtered image sequence is processed with a bank of 3D spatiotemporal Gabor filters of different spatiotemporal tuning frequencies for motion energy extraction. While traditionally this would have required *N*_S_^2^*N*_T_ times of multiply-and-accumulation (MAC) operations per pixel per frame (*N*_S_ and *N*_T_ are filter sizes along space and time dimensions, respectively), a separable implementation can be much more efficient. Given the fact that the horizontal or vertical components of the optical flow can be computed independently from the separate horizontal or vertical motion energy channels, the 3D spatiotemporal filter can be decomposed into cascaded spatial and temporal filters [14,25]. This way, the horizontal and vertical motion energy feature maps *ME*_X_ and *ME*_Y_ for different spatiotemporal tuning frequencies (*f*_X/Y_, *f*_T_) are extracted as:

(1) $$\begin{array}{l} I_{T}(x,y,t;f_{T})=I(x,y,t)*Gabor(t;f_{T}),\\ ME_{X}(x,y,t;f_{S},f_{T})=|I_{T}(x,y,t;f_{T})*Gauss(y)*Gabor(x;f_{X}){|}^{2},\\ ME_{Y}(x,y,t;f_{S},f_{T})=|I_{T}(x,y,t;f_{T})*Gauss(x)*Gabor(y;f_{Y}){|}^{2}, \end{array}$$

where * denotes convolution. The temporally filtered results *I*_T_ are reused in both the horizontal and vertical motion energy channels. With such a filter decomposition, the computational complexity in motion energy extraction reduces from *O*(*N*_S_^2^*N*_T_) to *O*(2 × 2 *N*_S_ + *N*_T_) [14,25]. The 1D spatial Gaussian and Gabor filters used in the two channels are identical but imposed along different spatial dimensions (*x* or *y*). The 1D Gaussian filter is defined as:

(2) $$Gauss(s)\approx \frac{1}{A_{0}}\exp(-\frac{s^{2}}{2\sigma_{S}^{2}}),$$

where *s* represents one spatial dimension (either *x* or *y*), standard deviation *σ*_S_ = *N*_S_, and *A*_0_ is a normalization factor ensuring all the filter weights summing up to 1. The 1D spatial and temporal Gabor filters in Equation (1) are complex-valued and defined as:

(3) $$Gabor(z;f_{Z})\approx Gauss(z)\exp(-i2\pi f_{Z}z),$$

where *z* represents one spatiotemporal dimension (*x*, *y* or *t*). The Gaussian window in Equation (3) is similar to the one defined in Equation (2), but with a standard deviation of *σ*_Z_ = 1/*f*_Z_.

### 2.2. Optical Flow Computation by Random Forests

The dense optical flow can be computed from the motion energy maps. The motion energy values of all the spatiotemporal tuning frequencies used in Equation (1) at a specific spatiotemporal point (*x*, *y*, *t*) is concatenated as feature vector **ME** = (*ME*(1), *ME*(2), …, *ME*(*n*))*^T^* for horizontal and vertical channels, respectively. The speed components at (*x*, *y*, *t*) can then be computed from the motion energy feature vectors in corresponding channels. In this work, we employ Random Forests (i.e., an ensemble of decision trees with higher accuracy than a single decision tree) to infer the speed values from the motion energy features. Compared to the weighted voting method [14,19] (also see Section 3.1 and Equation (6)) and other feasible methods (e.g., linear regression, relevance vector machine [29]), the Random Forests method is more suitable for low-cost real-time systems because in runtime it only involves a few simple operations (i.e., only comparisons and additions, no multiplications), and can still preserve its ability to handle sophisticated features in a nonlinear process. 

Random Forests is an ensemble of regression trees. The structure of one exemplar tree is shown in Figure 1c. The tree has two types of nodes: split nodes (disks) with two sub-trees, and leaf nodes (boxes) with no sub-trees. Each split node stores two parameters for splitting: a feature index *i* and a threshold *S*, and each leaf node has one parameter for prediction: the estimated motion speed *L* given by this leaf. The tree structure and parameters are learned from a large training (labeled) set of motion energy feature vectors with their true speed values. The trees are different from each other in structure and parameter values, due to randomness injected into the learning process of Random Forests (see details in [27]). The learning process is completed off-line on a PC before runtime. 

During runtime, a motion energy feature vector **ME** is processed starting from the root node (the topmost disk in Figure 1c) of each tree within the trained forest, and recursively passes down to left or right sub-tree depending on whether the indexed vector component *ME*(*i*) is less or greater than the threshold *S* for that node, until a leaf node is reached. At the leaf node of each tree, the predicted label *L* for that tree is obtained. The average of parameters *L* for all the trees of the Random Forests is the estimated speed for the input **ME** vector. Since the Random Forests predict only the magnitude of the optical flow and not the direction, the same set of trees can be reused for computing both horizontal and vertical motion speed components from the motion energy vectors in corresponding channels.

### 2.3. TTC Estimation from Optical Flow Field

The TTC map can be estimated from the dense optical flow **v** = (*v*_X_, *v*_Y_) [12] by calculating its divergence as shown below:

(4) $$\begin{array}{l} \mathrm{Div}(v(x,y))=\frac{\partial v(x,y)}{\partial x}+\frac{\partial v(x,y)}{\partial y}\\ =\frac{v_{X}(x+1,y)-v_{X}(x-1,y)}{2}+\frac{v_{Y}(x,y+1)-v_{Y}(x,y-1)}{2}, \end{array}$$

where Div(·) is the field divergence operator, and *v*_X_, *v*_Y_ are horizontal and vertical speed components of the optical flow. TTC can now be estimated from divergence as below:

(5) $$TTC(x,y)\approx 2/\mathrm{Div}(v(x,y))-N_{T}/2,$$

The term *N*_T_/2 is used to compensate for the response latency as the temporal Gabor filters used for motion energy extraction are symmetric Finite Impulse Response (FIR) filters with a latency of half of its length.

For more robust TTC estimation, two additional steps are incorporated. First, the optical flow is inaccurate at image locations with little texture that are associated with relatively lower motion energy (e.g., the region of white wall in an image). If the total amount of motion energy in the horizontal or vertical channel at a specific image location is below a threshold, the speed component in that channel is labeled as invalid for that location. The optical flow divergence and the TTC value at any given image location is labeled as invalid if any of the four speed components as required in Equation (4) is invalid. Second, the divergence map is spatially smoothed (average of all valid divergence in its *N*_S_ × *N*_S_ neighborhood) to achieve higher stability before calculating TTC by Equation (5). If more than a quarter of its neighborhood pixels have invalid divergence before smoothing, then the computed TTC at this location is also labeled as invalid. 

## 3. Experimental Results

For the experimental evaluation, the TTC estimation algorithm was implemented in Matlab 2017b. The following parameters were used. The DoG filter had a size of 41 × 41 pixels with a passband of 0.025 ~ 0.25 cycle/pixel. The 1D temporal Gabor filters had a length of *N*_T_ = 15 frames and their tuning frequencies were {0, ±0.05, ±0.1, ±0.15, …, ±0.4} cycle/frame. The 1D spatial Gaussian and Gabor filters had a size of *N*_S_ = 41 pixels, and the tuning frequencies of the spatial Gabor filters were {0.025, 0.05, 0.075, 0.1} cycle/pixel. The minimum threshold on the motion energy feature vectors to be considered valid as mentioned in Section 2.3 was set as 10^−5^. We adopted the Random Forests model provided in the Matlab machine learning toolbox with default parameters [30], except the number of regression trees was set as 20 and the maximum number of split nodes in each tree were set as 500. 

To train the Random Forests, we downloaded 30 (randomly chosen) natural scene images from the Internet and converted them to grayscale 320 × 240 format. Then we randomly chose an image and generated a sequence of *N*_T_ = 15 frames with a 2D translation motion pattern at a constant speed. The speed components were randomly chosen within the speed range of −8.5 to +8.5 pixel/frame with a uniform probability distribution. For more details regarding the motion generation, refer to [14]. Next, horizontal and vertical motion energy features were computed for 10 randomly chosen locations within the image sequence. Feature vectors above the motion energy threshold (10^−5^) were added to the training set. This procedure was repeated until 200,000 training samples were collected. Under the configuration of Random Forests as mentioned above, each tree trained with the 200,000 samples grew out to 500 split nodes and 501 leaf nodes, with a depth of 11. 

### 3.1. Optical Flow Accuracy

The accuracy of computed optical flow is critical to reliable TTC estimation. We compared the accuracy of optical flow computation using the Random Forests method to that using the weighted voting method described in [14]. For a fair comparison, the speed component candidates for voting were densely chosen from the range of −8 to 8 pixel/frame at a step of 0.1 pixel/frame, and the voting weight for a speed candidate *v* from a motion energy feature of spatiotemporal frequency pair (*f*_S_, *f*_T_) was pre-calculated as [19]:

(6) $$W(f_{S},f_{T},v)=\exp(-\frac{{\left(f_{S}v+f_{T}\right)}^{2}}{2\max(\varepsilon ,f_{S}^{2}v^{2}+f_{T}^{2})}),$$

where *ε* is a regularization term to prevent the divider in Equation (6) from being too small (otherwise, the voting would be too concentrated and not robust to noise). This term was empirically set as 0.01 through our simulation [14]. The candidate speed value receiving the most votes from all the motion energy units was the estimated speed component. 

**Figure 2:**
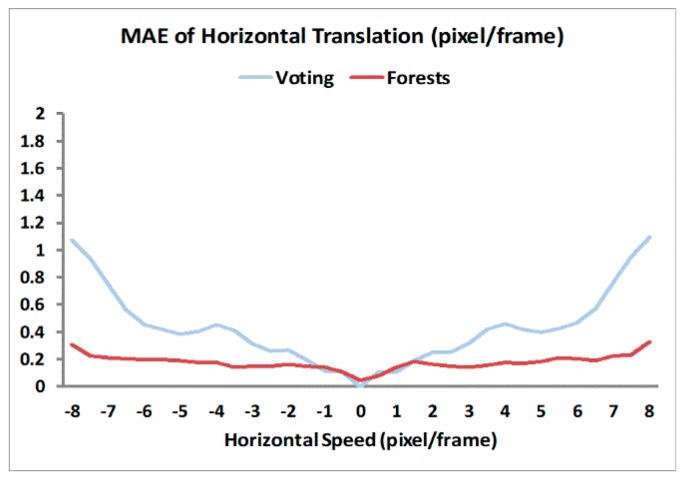
Mean Absolute Errors (MAE) of optical flow computation for 1D horizontal translation at different speeds from −8 to 8 pixel/frame with a step of 0.5 pixel/frame.

**Figure 3:**
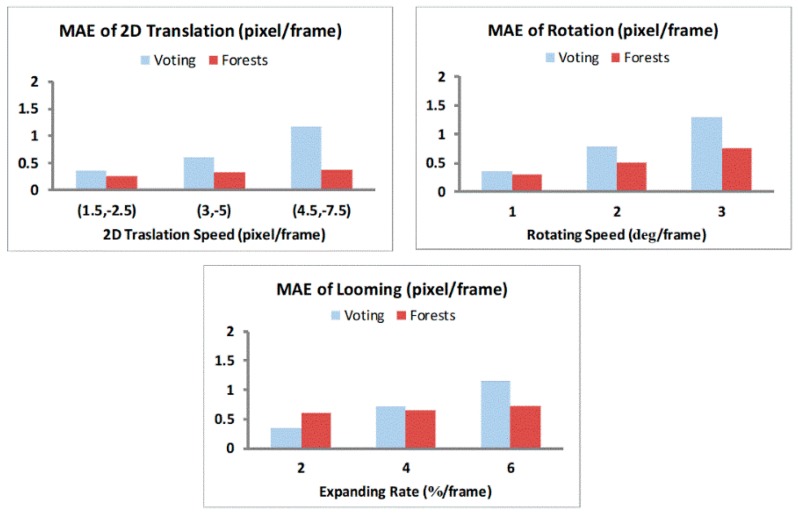
Mean Absolute Errors (MAE) of optical flow computation for more complicated motion patterns. In the looming pattern, each image frame is generated by expanding its preceding frame at a constant expansion rate, and then cropping the frame to leave only the central 320 × 240 region within the frame.

For optical flow computation, we converted the 12 Middlebury images [31] to grayscale 320 × 240 format to generate sequences with different motion patterns (1D/2D translations, rotations, and looming) [14]. Each sequence has a length of *N*_T_ = 15 frames. We compared weighted voting and Random Forests methods in optical flow computation, and sampled the results of every 10th pixel location along the horizontal and vertical dimensions on the optical flow maps. The Mean Absolute Errors (MAE) for each motion pattern across all the sampled locations in all relevant sequences are depicted in Figure 2 and Figure 3. For 1D horizontal translation, the absolute error of one pixel location is defined as the absolute difference between its computed and true horizontal speed values. For other motion patterns, the absolute error is defined as the distance (*L*2 norm) between its computed and true 2D speed vectors [31]. 

The horizontal speed values chosen for the 1D translation testing were from −8 to 8 pixel/frame at a step of 0.5 pixel/frame, thus totally 33 speed levels. For the other three testing patterns of 2D motion, there are three speed levels each. The average MAEs for voting and Random Forests methods under each motion pattern over their respective speed levels are shown in Table 1. 

For the 1D horizontal translation pattern, voting and Random Forests methods had comparable accuracy at lower speed magnitudes (<1 pixel/frame), but the MAE for the voting method increased and was higher than Random Forests method for speed magnitudes upwards of 2 pixel/frame with a drastic increase at higher speeds (>6 pixel/frame). In contrast, the error of Random Forests was approximately constant for speeds from 1 to 7.5 pixel/frame, and only slightly increases when the speed approaches 8 pixel/frame. Figure 3 further confirms these observations as we see lower MAE values for the Random Forests method compared to the voting method for rotation and looming sequences. These results demonstrate that Random Forests achieved much higher accuracy in optical flow at moderate- to high-speed motion because of their ability to adapt to different speed values obtained from the learning stage. Such an adaptation is absent in the voting method with a fixed set of voting weights, which cannot be optimally tuned for different speed levels. Overall, the results demonstrate that the biological motion energy features along with the Random Forests can compute optical flow with low errors, which has a major consequence when computing TTC.

### 3.2. TTC Estimation Results

**Figure 4:**
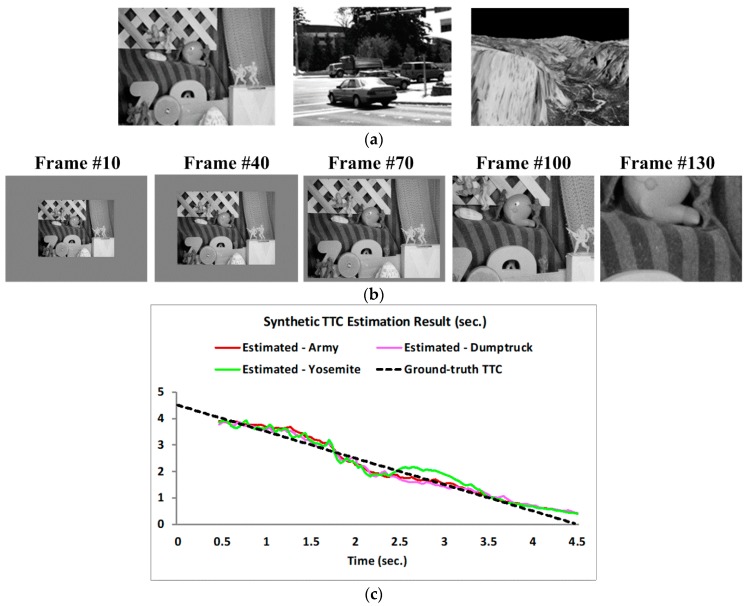
TTC estimation results for synthetic looming sequences. (**a**) Three Middlebury images selected to generate synthetic looming sequences. (**b**) One of the synthetic looming sequences: the Army looming sequence. (**c**) Global averages of estimated TTCs of the three sequences.

We tested the proposed TTC estimation algorithm with both synthetic and real image sequences. Synthetic looming sequences simulated constant velocity approaching camera with TTC range of 5 down to 0.5 s (there were totally 135 frames for a 30 fps camera, and TTC values between this range were uniformly assigned to the frames as ground truth). They were also created using three Middlebury images: Army, Dumptruck and Yosemite, as shown in Figure 4a. In each sequence, the first frame was rescaled to 160 × 120 and wrapped with a constant grayscale background to form a 320 × 240 resolution, as shown in Figure 4b. We assumed a linear TTC decrease from 4.5 s down to 0 s and the frame rate of synthetic sequences was 30 fps. So each frame was labeled with a unique TTC value. For example, the 15th frame corresponded to a TTC value of 4.5 – (15/30) = 4 s. The expansion rate (i.e., optical flow divergence) at each frame time was calculated from the corresponding TTC value based on Equation (5). And then the expanded frame was depicted following the expanding optical flow. Figure 4c shows the simulation results on the three synthetic looming sequences. The TTC plots show average estimated TTC values and the ground truth over time (frames of the sequence converted to time using frame rate of 30). The average estimated TTC value was computed over the entire frame for all the valid estimates. Since the image was simulated to be looming as a whole, such global average is reasonable and provides a quantitative way of evaluating the algorithm performance. The results in the first 0.5 s were not computed as the temporal Gabor filter in the proposed algorithm needed a 15-frame length (0.5 s under 30 fps) for filtering. We can see that the TTC plot matches well with the ground truth (GT) with an RMS difference of 0.210 s for all three sequences over the whole looming course. 

**Figure 5:**
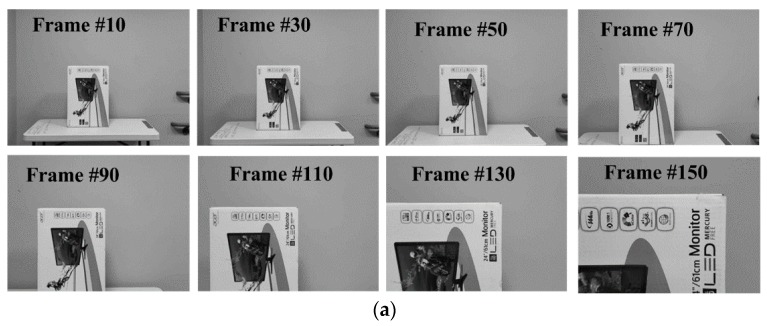

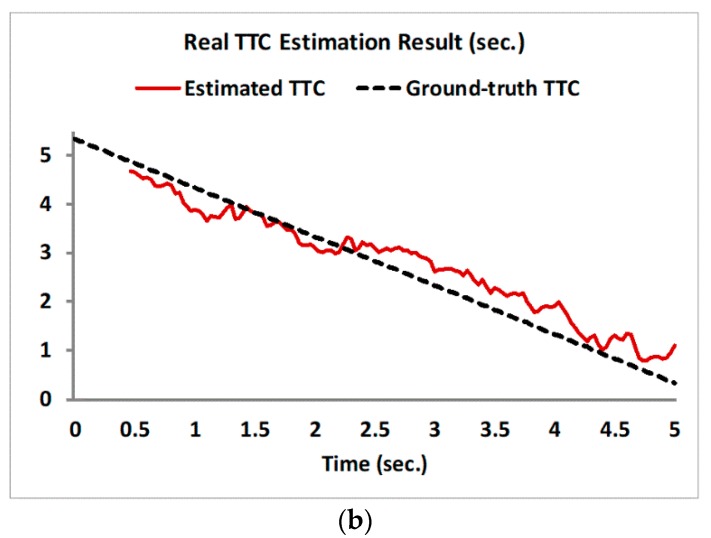
TTC estimation results for a real-world looming sequence. (**a**) Image sequence captured from a moving camera approaching a stationary object. (**b**) Global average of estimated TTC for obstacle in the sequence.

We also tested our algorithm on a real-world sequence captured by a 30 fps camera moving toward a stationary obstacle at a constant pace. The expected TTC results should approximately linearly decrease during its motion. Since the sequence had only a single obstacle, we again computed the average of all valid TTCs on the maps, representing a unified region-level TTC value for the object. Figure 5 shows the plot of estimated global TTC along with some recorded frames. The resulting plot reflects the decreasing trend in TTC as expected and the RMS difference between the estimated TTC and the ground truth over the entire looming sequence was 0.365 s. 

**Figure 6:**
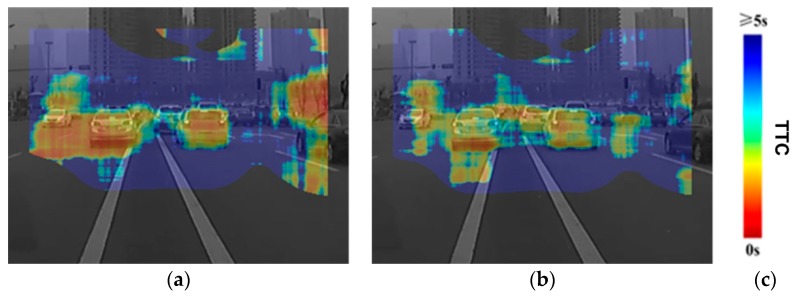
The TTC map sequence for a real-world driving video clip, overlaid on the image frames. The video was captured with a dashboard camera from a car, initially moving at a velocity of about 20 km/h and then decelerating and coming to a complete halt. Colors trending toward red show shorter TTC values, whereas those trending toward blue show longer TTC values. TTC values longer than 5 s are shown as blue. (**a**) far from the observing car. (**b**) a little closer to the observing car at slightly decelerated velocity. (**c**) legend bar for TTC heat map values.

Finally, we show results of the proposed TTC algorithm for a more challenging real-world driving video clip containing multiple independently moving objects as well as camera self-motion (Figure 6). The TTC heatmap (clamped at 5 s – colors trending toward red show lower TTC values) is overlaid on the input image frames. The regions (including the border) without heatmap colors overlaid indicate the computed TTC values for them are invalid and thus not shown. As an overview, the scene contains many objects at a variety of depths and shows the car with the on-board camera braking as it approaches stopped or slowing traffic in front. For Figure 6a, we can clearly see that the two cars on the front have approximately the same TTC, which is as expected. The oncoming car on the far left side of the image is farther from the camera, but has approximately the overall TTC values as the car directly in the front because of its approaching speed. This is again as expected. Figure 6b shows another frame further along the sequence, where the oncoming car has mostly passed the processing zone and thus the TTC map only covers the rear portion of the car on the extreme left of the TTC map. As the car with the camera has also slowed down further, the TTC values are trending toward larger values compared to Figure 6a. In both cases, large parts of the background, including the buildings, show very high TTC values.

## 4. Discussion

The random forest is trained using spatiotemporal features rather than the image content itself. Our training image set was real-world pictures selected from the internet, and it is well known that real world images share the 1/f characteristic in the power spectrum. Therefore, the trained decision trees used in our experiment can generalize well to other applications involving natural images, unless the image spectrum is very different, for instance, in man-made environment dominated by vertical edges. In those scenarios, the decision trees of the Random Forests algorithm may need to be re-trained with images representative of that particular scenario. 

**Figure 7:**
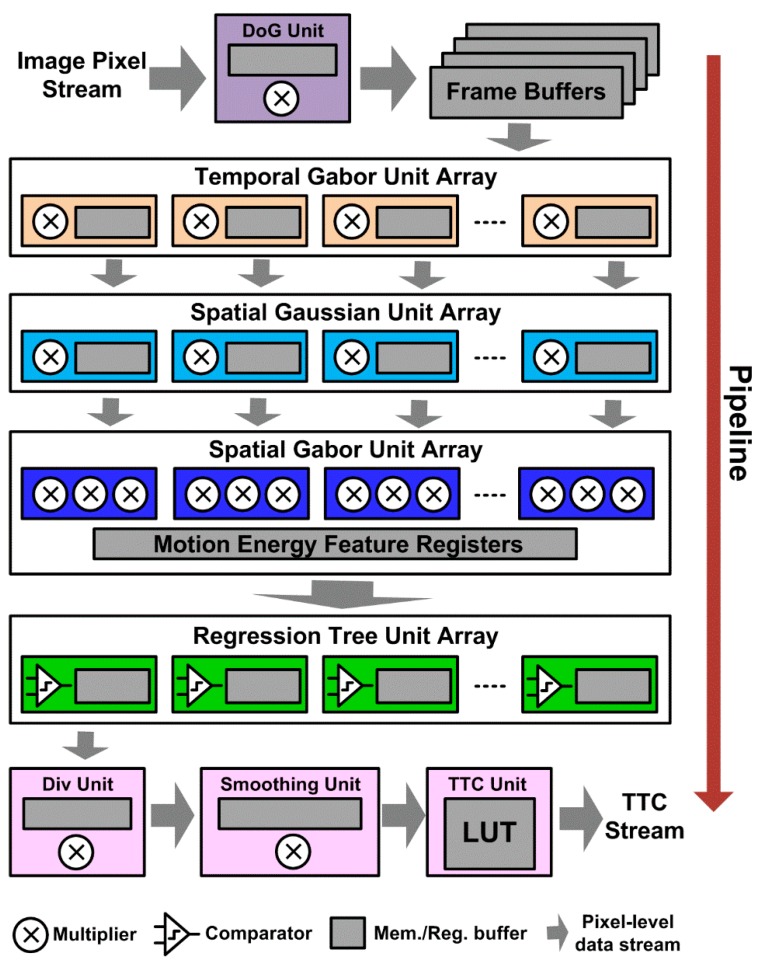
The conceptual diagram of pixel-stream pipeline hardware architecture for our TTC estimation algorithm.

Our TTC estimation algorithm is well-suited for low-cost real-time embedded systems typically deployed on smart video sensors. It can readily be implemented with a pixel-stream multiple-level pipelined architecture (see Figure 7) similar to that in [14]. Such architecture has good scalability to allow flexible tradeoffs among estimation accuracy, processing speed, and resource budget. For example, we can cut down spatiotemporal tuning frequency points and save the resources associated with the corresponding Gabor filters at the cost of less representative motion energy, or we could add more decision trees to improve accuracy of optical flow. We could reuse the same set of spatial Gabor filters and Random Forests for computing both horizontal and vertical speed components in a time-multiplexing mode, or duplicate another such set to process the two channels in parallel to achieve higher system throughput. In this architecture, the structure and circuit designs of those blocks for motion energy extraction (e.g., the Gabor filter arrays) will be the same as those in [14]. The expensive massively parallel speed voting array in [14] can be replaced by a low-cost array of parallel regression tree blocks. With the parameter configuration used in Section 3, the voting array would consume 161 multipliers for MAC operations and 161 × 17 × 4 × 16 b = 21.4 kB memory capacity to store the 16-bit voting weights. In Random Forests, each split node needs to store 3 parameters: feature index (8-bit), split threshold (24-bit) and the memory address (12-bit × 2) of its left and right sub-nodes. Each leaf node needs to store one parameter: the prediction value (12-bit). So the Random Forests method would require ((8 b + 24 b + 12 b × 2) × 500 + 12b × 501) × 20 ≈ 85 kB memory space, given the tree properties in Section 3. However, the decision tree traversing involves only simple operations: memory access, comparisons, and additions, which require vastly fewer computational resources. Importantly, no multipliers are needed. The division for tree prediction averaging can be approximated by additions since the number of trees (*M*) is a constant in runtime and × (1/*M*) can be pre-calculated and approximately decomposed into a series of negative powers of 2. Thus the division can be done via bit-shifting and addition operations. For instance, when *M* = 20, then the multiplication operation of *A* × (1/20) can be approximately decomposed as *A* × (1/16 – 1/64 + 1/256) = (A>>4) – (A>>6) + (A>>8). Since memory banks are much cheaper than computing units on modern nanoscale devices, the memory-centric Random Forests is much more economic for hardware implementation than the voting method. Moreover, Random Forests can run much faster than the voting array. For each parallel voting unit in [14], it requires at least 68 clock cycles to accumulate the votes from all 17 × 4 motion energy units for one pixel. In contrast, for each parallel tree unit with a depth of 11 as used in Section 3, it requires only 2 × 11 = 22 clock cycles for a motion energy vector to reach its leaf node (one split node consumes two cycles: one for memory access to fetch node parameters, another for feature indexing, comparison and branching). The operations of winner search in voting array and tree prediction averaging in Random Forests can be pipelined in parallel with vote accumulation and tree traversing, respectively. So their time consumption is not counted in this calculation. Lastly, a few more units for computing TTC from optical flow will need to be appended to the architecture. The divergence computation and smoothing operations are essentially image filtering, and can be easily realized as any other spatial filter unit. The division operation in (5) can be realized via a small memory-based lookup table (LUT). For 0.5-5 s TTC range with 0.1 s decimal precision, the length of LUT will be only 46. 

Generally, our TTC method can be implemented on a hardware platform with low to moderate resource cost, such as the Zynq-7045 or Kintex-7 serial FPGA. Using a pipeline architecture similar to that employed in [14] and with the pipeline bottleneck removed by replacing the voting block in [14] with the much faster forests module as discussed above, our future hardware system is estimated to reach the 30 fps real-time performance on a 320 × 240 resolution. For higher resolutions above 320 × 240, we can add a simple downsampling stage before DoG filtering to maintain the real-time processing speed, just as did in [14]. Note the processing units in each array module are processing in parallel, so increasing the numbers of these units would not contribute to processing speedup but only to estimation accuracy improvement. The selection of the number of units depend on the tradeoff between the resource budget and the required accuracy for the given application. 

## 5. Conclusions

In this paper we have shown that the proposed TTC estimation algorithm is accurate, hardware friendly, and can be potentially implemented on a smart video sensor hardware. The algorithm estimates TTC from dense optical flow based on biological motion energy features and Random Forests. The results demonstrate that the Random Forests method helps to achieve higher optical flow accuracy and consumes less computation resources compared to the voting method in previous works. Our TTC estimation algorithm is hardware-friendly and can readily be implemented in a pixel-stream pipelined scalable architecture allowing for flexible tradeoffs among estimation accuracy, processing speed and resource consumption. Deployment and testing on a smart video sensor are our future work. 

## Figures and Tables

**Table 1:** The average MAEs (lower values indicate better accuracy) of the 4 different motion patterns across their respective speed levels.

Avg MAE (pixel/frame)	Horizontal Translation	2D Translation	Rotation	Looming	Global
Voting	0.435	0.712	0.815	0.740	0.676 ^1^
Forests	0.179	0.320	0.519	0.658	0.419 ^1^

^1^ Computed by averaging the data in the previous four columns.
